# Interaction of job-related psychological flexibility, coping style and personality types in depression in Chinese physicians: A cross-section study

**DOI:** 10.1097/MD.0000000000030838

**Published:** 2022-09-30

**Authors:** Yongcheng Yao, Xiangzhi Jing, Lingeng Lu

**Affiliations:** a Zhengzhou Normal University, Zhengzhou, Henan, China; b Xinyang Vocational and Technical College, Xinyang, Henan, China; c Department of Chronic Disease Epidemiology, Yale School of Public Health, Yale University, New Haven, CT, USA.

**Keywords:** coping style, depression, job-related psychological flexibility, personality type, physicians

## Abstract

To investigate the associations of job-related psychological flexibility, coping style and personality types with and their interactions in depression in Chinese physicians. A cross-sectional survey of 444 physicians was conducted by using the convenience sampling method in the municipal hospitals in Zhengzhou, Henan province. Center for Epidemiological Studies Depression, Work-related Acceptance and Action Questionnaire, the Simplified Coping Style Questionnaire and Eysenck Personality Questionnaire-Revision Short Scale of China were administered to each participant. Depression tendency scores were significantly higher in healthcare workers with intermediate title, age 31 and older, introvert unstable personality than other counterparts, (*P < *.01). Female and extrovert stable healthcare workers had significantly higher coping score than male and other personality types (*P < *.05). The scores of job-related psychological flexibility in healthcare workers with Introvert Stable or working in emergency department were significantly higher than their counterparts (*P < *.01). General linear model algorithm of machine learning showed that Extrovert Unstable was the main risk factor for depression (*β = *6.74), followed by Extrovert Stable (*β = *−4.90), negative coping, positive coping, and length of service. Multivariate regression models showed that a significant interaction existed between coping style, work-related psychological flexibility and Extroversion (*β *= −0.103, *P < *.05), independently explaining 0.7% variance of depression, and that a significant interaction existed between coping style, work-related psychological flexibility and neuroticism (β = 0.116, *P* < .05), independently explaining 1.0% variance of depression. Interactions existed between personality types, coping style and work-related psychological flexibility in depression tendency in Chinese healthcare workers, with neuroticism (extrovert unstable) being a risk factor and extroversion (extrovert stable) being a protective factor. Precision prevention strategies could be made based on personality types to reduce depression in health workers.

**Current research questions**

- ► What is the prevalence of depression disorders in Chinese physicians?
- ► What is the impact of mental health literacy program on Chinese physicians?
- ► Is there the influence of job-related psychological flexibility on Chinese physicians’ mental health?

**Strengths and limitations of this study**

- ► The study sample size is relatively large with 444 Chinese physicians included
- ► This is the first study to investigate the associations of job-related psychological flexibility, coping style and personality types with and their interactions in depression in Chinese physicians.
- ► General linear model algorithm of machine learning shows that Extrovert Unstable is the main risk factor for depression.
- ► Significant interactions existed between coping style, work-related psychological flexibility and personality.
- ► Limitation is that this is a cross-sectional study, and further cohort studies are needed to determine the causal relationship. Convenience sampling may have some bias, and the results may not be able to be generalized to population.

## 1. Introduction

Depression is a common and serious mental disorder, which is characteristic of feeling sad and having a depressed mood.^[1,2]^ Depression negatively affects one’s feeling, thoughts and action, leading to loss of interest or pleasure in activities, alteration of appetite, aberrant sleeping, feeling fatigue, even tendency to suicide.^[1,2]^ The global burden of depression increases by 49.86% during the period of 1990 and 2017.^[3]^ It has been shown that depression occurs in 52.4% of professional healthcare workers in China,^[4]^ suggesting that healthcare workers are one of the groups at a higher risk of depression than the general population. Its prevalence in China is much higher than in high-income nations (range between 21.53% and 32.77%), and in the general population worldwide (4.40% in 2015).^[5–7]^ A recent meta-analysis study showed that the prevalence of depression was 33.03% (95% CI: 27.40–29.19%) in healthcare workers in the Eastern Mediterranean Region.^[8]^ Healthcare workers with depression may negatively affect the quality of their service to their patients, leading to patient dissatisfaction, medical mistakes, and associated financial costs.^[9–11]^ In some severe cases, patient safety may be compromised.^[12]^ Besides the general stressors in their personal lives, healthcare workers usually also face to exceedingly high levels of academic and professional stress, which results in emotional exhaustion and burnout.^[13–15]^ Study shows that depression is an important determinant of exhaustion of burnout.^[16]^

Job-related psychological flexibility (JPF) refers to one’s ability to persist with acceptance behavior in pursuit of goals and values and to engage the work no matter how painful thoughts and feeling, such as stress and anxiety that an individual experiences.^[17]^ Job-related psychological flexibility is a protective factor for several mental disorders including depression.^[18–20]^ Individuals who have high job-related psychological flexibility can help physicians take advantage of the beneficial resources in the work environment and better adapt themselves to the work environment.^[21,22]^ Individuals can still work efficiently even when they face stress in the workplace. The attitude to the stress also depends on one’s coping style.^[23]^ Individuals with a positive coping style like to directly face the stress and solve problem, enabling ones to embrace the external social environment. In contrast, individuals with a negative coping style prefer to escape or avoid, emotionally change behavior, and feel anxious and depressed.^[24]^

Personality is the overall psychological characteristics of an individual in thinking, feeling and behaving. It has 2 dimensions, sociability (extrovert or introvert) and emotional stability (irritable, impulsive and sensitive or not),^[25]^ which was narrowly defined for the purpose of this study with the guidance of the survey tools chosen for this study. Personality is exhibited by interacting with each other in the social environments, and is defined as psychoticism, neuroticism (negative emotionality), introversion and extroversion.^[26]^ It has been shown that different personality types have an impact on the degree of depression, with introvert having a negative effect on the depression.^[27]^ Accumulating evidence suggest that depression is linked to personality such as neuroticism/negative emotionality, extroversion/positive emotionality.^[28]^ We previously reported that personality types were associated with burnout in Chinese healthcare workers,^[13]^ which is related to depression. These previous observations suggest that job-related psychological flexibility, coping styles and personality types may associate with depression and their interactions exist in depression. To address this scientific issue, we conducted a cross-section study in China, and applied a convenience sampling approach.

## 2. Participants and Methods

### 2.1. Participants

The convenience sampling method was applied in this study based on the principal of “willing and agree” to participate in the study. During the period from June to August, 2015, we conducted a cross-section survey on physicians in municipal hospitals in Zhengzhou, the capital of Henan Province, China. The inclusion criteria include: working length >1 year, and able to work normally without physical and/or mental illnesses. The informed consent was obtained from all participants. The protocol for this study was reviewed and approved by the institutional research committee of Zhengzhou Normal University. All procedures performed in this study involving the participants are in accordance with the 1964 Helsinki declaration and its later amendments or comparable ethical standards.

### 2.2. Methods

All the questionnaires were administered to each participant who agreed to participate in this study. In the introduction part of the questionnaires, the demographic information include age, sex, marital status, length of service (working length), job title, education, and department.

#### 2.2.1. Patient and public involvement

No patients but professional healthcare workers were involved in this study. A cross-sectional study was conducted with the enrollment of 444 healthcare workers in the hospitals of Zhengzhou.

#### 2.2.2. Depression tendency evaluation

The frequency of depression tendency was assessed on the participants using Center for Epidemiological Studies Depression.^[29,30]^ The scale includes 4 dimensions of somatization symptoms, depression, positive emotions and interpersonal problems, with a total of 20 items. A 4-level scoring method (range from 0.0 to 3.0 points) is adopted, and the sum of the scores of all items is the total score of the scale; the higher the total score is, the more severe the depression tendency indicates. The Cronbach’s α coefficient of this scale is 0.957.

#### 2.2.3. Job-related psychological flexibility

The Work-related Acceptance and Action Questionnaire was used to evaluate job-related psychological flexibility.^[31]^ There are 7 items each with Likert scale 1 to 7 in the questionnaire in Chinese version, which has been shown high reliability and validity.^[32]^ The sum score of the items reflects the extent of work acceptance. A higher score indicates a higher job-related psychological flexibility and a higher work acceptance. The Cronbach’s α coefficient of this scale is 0.863.

#### 2.2.4. Personality types

The personality type was determined using the version of the Eysenck Personality Questionnaire in Chinese, which is translated and revised by Qian Mingyi, a professor of psychology at Peking University.^[33]^ Reliability and validity of the scale has been evaluated with high quality, and it is suitable for testing in Chinese. It has been widely used in medicine, justice, education and other fields for personality survey. Two dimensions, introversion-extroversion (E) and neuroticism-emotional stability (N), were used to assess the personality type. The neuroticism/stability scale (EPQ-N) was for emotional stability when facing negative affect such as depression and anxiety, and the introversion and extroversion vector scale (EPQ-E) for the demand of external stimulation. There are a total of 24 items each with a score of either 0 or 1. A low sum score in the E dimension indicates an introversion, whereas a high sum score indicates an extroversion. A low sum score in the N dimension indicates emotional stability. In contrast, a high sum score indicates emotional instability. Based on the score cutoffs of 43.3 and 56.7 for the E and N dimensions, respectively, 4 personality types were defined: stable introvert, stable extrovert, unstable introvert, and unstable extrovert. The Cronbach’s α coefficient of the EPQ-E and EPQ-N in this study was 0.637 and 0.805, respectively.

#### 2.2.5. Coping style

A simple coping style questionnaire was applied to assess the coping style of the participants.^[34]^ This questionnaire is composed of 20 items, which measure 2 dimensions: positive coping and negative coping. The positive coping dimension reflects the characteristics of positive coping such as “seeing the good side of things as much as possible.” In contrast, the negative coping dimension reflects the negative coping characteristics such as “fantasy that some miracle may happen to change the status quo.” The questionnaire is a self-evaluation scale with 4 grades. Participants choose a good answer based on their own situation. The result is the difference between the average score of the positive coping dimension and the average score of the negative coping dimension. The Cronbach’s α coefficient of this scale is 0.868.

### 2.3. Statistical analyses

Statistical analyses were performed using SPSS 18.0 software. Normality test (the absolute values of Skew and kurtosis are both less than 1) was applied to check the distribution of the numeric variables first to choose appropriate statistics and statistical analysis methods. The Bonferroni method was used for the correction of multiple comparisons, and the Dunnett T3 method was used when the variance was uneven. The generalized linear algorithm (GLM) in the machine learning was performed to analyze the importance of factors in depression tendency in H2O.ai (http:/docs.h2o.ai/h2o/latest-stable/H2O docs/flow.html). Pearson correlation analysis was performed on physicians’ depression tendency, job-related psychological flexibility, coping style and personality types. Using the ModGraph,^[35]^ the interactive relationship diagram between depression tendency, job-related psychological flexibility, coping style and personality types was constructed,^[36]^ and multiple regression analysis was used to explore the ternary interaction effect of job-related psychological flexibility, coping style and personality types on depression tendency. A *P* value <.05 is considered statistically significant.

## 3. Results

### 3.1. The characteristics of the participants

A total of 500 questionnaires were distributed, and 444 valid questionnaires were returned. The response rate was 88.6%. Among the 444 participants, 175 are males (39.4%) and 269 females (60.6%). The average age was 35 years old with the range of 20-60 years old. The average length of service (working length) was 11 years with the range between 1 and 44 years. Of the participants, 127 (28.6%) physicians work in internal medicine, 127 (28.6%) in surgery, 50 (11.3%) in obstetrics and gynecology, 36 (8.1%) in emergency department (8.1%), and 104 (23.4%) in other departments.

### 3.2. Common-method variance (CMV) test

The Harman’s single factor test was used to measure the CMV. The result showed no severe CMV existing in this study. Twenty-four factors had eigenvalues greater than 1, and 16.93% of the variance was explained by the first factor, which is less than the criterion of 40%.^[37]^

### 3.3. Associations of depression tendency, coping style and job-related psychological flexibility

Table 1 shows the associations of demographic factors with depression tendency, coping style and job-related psychological flexibility in Chinese physicians. The coping style score was significantly higher in female physicians than male (*P < *.05). There was a significant association between age and the score of depression tendency (*P < *.01). Further pairwise comparison found that the score of depression tendency in physicians aging 31 to 35 years old was significantly higher than those in the greater than 36 years old group (*P < *.01). Professional title was also found in a significant association with the score of depression tendency (*P < *.05), with the intermediate professional title having higher than other professional titles (*P < *.01). Personality types were significantly associated with the scores of depression tendency, copying style, and job-related psychological flexibility (*P < *.001). Physicians with introvert unstable personality had the highest depression tendency, followed by extrovert unstable, introvert stable and extrovert stable personality, respectively. In contrast, physicians with extrovert stable personality showed the highest coping abilities, followed by introvert stable, extrovert unstable and introvert unstable personality, respectively. Physicians with introvert unstable personality had the lowest positive coping scores, whereas those with extrovert stable personality had the highest positive coping scores. Physicians with extrovert stable personality had a significantly higher job-related psychological flexibility than those with other personality types. A significant association was also found between the working length and the positive coping style score (*P < *.05), with the long working length having a higher positive coping style score. There was a significant difference in job-related psychological flexibility between the departments (*P < *.01). Physicians working in emergency department showed a higher job-related psychological flexibility than those working in other departments (*P < *.01). No significant associations were found between either marital status or education and either depression tendency, coping style, positive and negative coping style scores, or job-related psychological flexibility.

**Table 1:** Associations of demographic variables with depression tendency, coping style and job-related psychological flexibility (mean ± SD).

**Variable**	*N*	**Depression** **tendency**	**Coping style**	**Positive coping**	**Negative coping**	**JPF**
**Sex**						
Male	175	16.71 ± 12.53	0.50 ± 0.69	2.86 ± 0.59	2.36 ± 0.64	34.38 ± 8.42
Female	269	18.58 ± 10.38	0.67 ± 0.67	2.95 ± 0.56	2.28 ± 0.52	33.84 ± 7.64
*t*		−1.641	−2.579^*^	−1.665	1.332	0.686
**Marital status**						
Single	85	18.11 ± 11.12	0.61 ± 0.68	2.85 ± 0.59	2.24 ± 0.50	33.24 ± 8.25
Married	359	17.79 ± 11.35	0.60 ± 0.68	2.93 ± 0.57	2.33 ± 0.58	34.24 ± 7.88
*t*		0.235	0.164	−1.103	−1.318	−1.050
**Age**						
<31	157	17.68 ± 9.94	0.62 ± 0.67	2.90 ± 0.59	2.28 ± 0.55	33.80 ± 7.41
31~	130	20.35 ± 12.18	0.51 ± 0.67	2.85 ± 0.57	2.34 ± 0.61	33.42 ± 8.07
36~	157	15.94 ± 11.50	0.66 ± 0.70	2.98 ± 0.57	2.33 ± 0.56	34.82 ± 8.35
*F*		5.560^**^	1.594	1.904	0.387	1.231
**Working Length (yrs**)						
1~	167	18.48 ± 10.44	0.58 ± 0.68	2.88 ± 0.57	2.29 ± 0.57	34.11 ± 7.26
6~	118	19.15 ± 11.45	0.54 ± 0.62	2.84 ± 0.57	2.30 ± 0.57	32.87 ± 8.12
11~	159	16.21 ± 11.92	0.67 ± 0.72	3.01 ± 0.58	2.34 ± 0.57	34.86 ± 8.45
*F*		2.731	1.308	3.426^*^	0.304	2.125
**Job title**						
Primary	192	17.19 ± 10.27	0.61 ± 0.67	2.88 ± 0.60	2.27 ± 0.53	34.51 ± 7.83
Intermediate	163	20.72 ± 12.17	0.55 ± 0.71	2.91 ± 0.56	2.36 ± 0.61	32.87 ± 7.74
Senior	89	14.00 ± 10.48	0.67 ± 0.65	3.00 ± 0.57	2.32 ± 0.57	35.21 ± 8.40
*F*		11.269^***^	0.928	1.289	1.166	3.095^*^
**Education**						
college degree or below	39	17.21 ± 11.44	0.64 ± 0.69	2.94 ± 0.57	2.30 ± 0.51	34.74 ± 6.90
bachelor degree	231	17.62 ± 11.34	0.62 ± 0.70	2.94 ± 0.62	2.31 ± 0.57	34.12 ± 8.49
master degree or higher	174	18.29 ± 11.26	0.56 ± 0.66	2.88 ± 0.51	2.32 ± 0.59	33.80 ± 7.44
*F*		0.239	0.561	0.562	0.027	0.239
**Personality type**						
Introvert Stable	25	14.52 ± 7.64	0.63 ± 0.47	2.80 ± 0.58	2.17 ± 0.52	32.64 ± 9.00
Introvert Unstable	52	28.46 ± 11.46	0.18 ± 0.65	2.55 ± 0.55	2.37 ± 0.43	31.04 ± 8.00
Extrovert Stable	55	7.09 ± 7.19	1.05 ± 0.71	3.30 ± 0.50	2.25 ± 0.65	40.35 ± 6.35
Extrovert Unstable	24	25.79 ± 10.50	0.35 ± 0.53	2.86 ± 0.55	2.51 ± 0.64	32.46 ± 5.56
*F*		52.750^***^	18.202^***^	17.739^***^	1.890	16.743^***^
**Department**						
Emergency	36	19.56 ± 13.00	0.53 ± 0.66	2.89 ± 0.60	2.36 ± 0.63	37.89 ± 7.34
Surgical	127	17.40 ± 11.74	0.60 ± 0.74	2.90 ± 0.59	2.31 ± 0.59	34.41 ± 7.61
Obstetrics and gynecology	50	16.16 ± 10.69	0.63 ± 0.75	2.91 ± 0.65	2.28 ± 0.46	34.22 ± 9.34
Medicine	127	19.20 ± 10.22	0.59 ± 0.60	2.87 ± 0.52	2.28 ± 0.57	32.48 ± 7.15
Other	104	16.96 ± 11.64	0.63 ± 0.69	2.99 ± 0.59	2.36 ± 0.56	34.12 ± 8.40
*F*		1.148	0.170	0.688	0.423	3.484^**^

Coping style score is the sum of positive and negative coping scores.

JPF = job-related psychological flexibility.

*P < .05, **P < .01, ***P < .001.

### 3.4. GLM analysis of risk factors in physicians’ depression tendency

**Figure 1.**
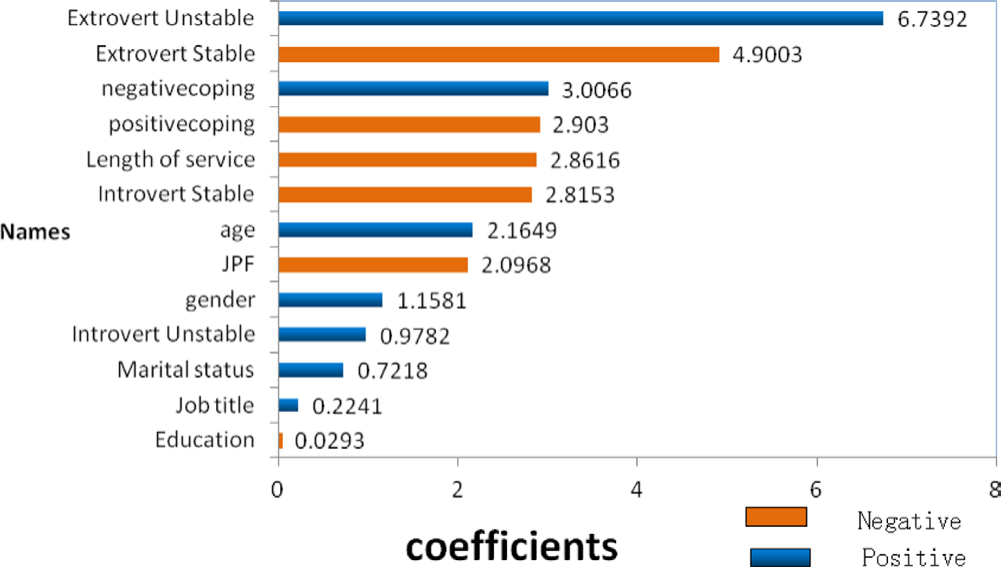
Importance of risk factors in depression tendency. Blue bars indicate a positive association with depression tendency, and orange bars indicate a negative association with depression tendency.

In order to explore the importance of risk factors in physicians’ depression tendency, we performed the GLM algorithm in H2O machine learning (H2OFlow of H2O.ai). The results are shown in Figure 1 (blue represents a positive association, orange represents a negative association). Extrovert unstable personality ranked the top among the risk factors of physicians’ depression tendency (standardized coefficient = 6.74), followed by the extrovert stable (standardized coefficient = −4.90), negative coping style (standardized coefficient = 3.01), positive coping style (standardized coefficient = −2.90), working length (standardized coefficient = −2.86), introvert stable (standardized coefficient = −2.82), age (standardized coefficient = 2.16), JPF (standardized coefficient = −2.10), sex (standardized coefficient = 1.16), introvert unstable (standardized coefficient = 0.98), marital status (standardized coefficient = 0.72), job title (standardized coefficient = 0.22), and education (standardized coefficient = −0.03). Extrovert unstable personality, negative coping style, age, sex, introvert unstable, marital status and job title are positively correlated with depression tendency. In contrast, extrovert stable personality, positive coping style, working length, JPF and introvert stable personality are negatively correlated with depression tendency.

### 3.5. Correlation between job-related psychological flexibility, coping style, personality types and depression tendency

Table 2 shows the correlations between job-related psychological flexibility, coping style, personality types and depression tendency. Pearson correlation results demonstrated that a significantly positive correlation between neuroticism (emotional stability) and depression tendency (*R *= 0.608，*P < *.01). A significantly negative correlation was also found between depression tendency and either coping style, job-related psychological flexibility, or extroversion. The correlation coefficients were −0.413, −0.347, −0.336, respectively (*P < *.01).

**Table 2:** Pearson correlations between the variables (n = 444).

**Variable**	***M***	***SD***	**Depression**	**Coping style**	**JPF**	**Extroversion**
Depression	17.85	11.30	1.000			
Coping style	0.60	0.68	−.413*	1.000		
JPF	34.05	7.95	−.347*	.343*	1.000	
Extroversion	7.29	2.82	−.336*	.260*	.267*	1.000
Neuroticism	5.46	3.30	.608*	−.344*	−.285*	−.262*

M = mean, SD = standard deviation. JPF = job-related psychological flexibility; neuroticism: emotional stability.

* *P* < .01.

### 3.6. Interaction of job-related psychological flexibility, coping style and personality types in depression tendency

Three different multivariate regression models were constructed to investigate the associations of job-related psychological flexibility, coping style and personality types with depression tendency, and the results are illustrated in Table 3. In the model 1, the main effects of job-related psychological flexibility, coping style and emotional stability (personality types) only were included. The 3 variables explained 25.3% of the variance of depression tendency. All 3 variables showed significantly negative associations with depression tendency (*P < *.001), with coping style having the largest weight (*β = *−0.293). The model 2 included the 2-way interaction terms of job-related psychological flexibility, coping style, extroversion personality beyond the main effects. The 3 main effects remained significant. However, all 3 2-way interaction terms were not statistically significant (*P* > .05). In the Model 3, we further added one 3-way interaction of job-related psychological flexibility, coping style and extroversion personality beyond the model 2. The 3-way interaction term was found statistically significant (*β = *−0.103, *P* = .044), which independently explained 0.7% of the variance of depression tendency. The main effect of coping style was the greatest with a negative coefficient (*β = *−0.266, *P < *.001).

**Table 3:** Associations of WPF, extroversion and coping style with depression tendency.

**Variable**	Model 1		Model 2		Model 3	
	β	*P* value	β	*P* value	β	*P* value
Coping style	−0.293	<.001	−0.291	<.001	−0.266	<.001
WPF	−0.191	<.001	−0.194	<.001	−0.176	<.001
E	−0.209	<.001	−0.201	<.001	−0.168	<.001
Coping style × WPF			−0.004	>.05	0.008	>.05
Coping style × E			0.012	>.05	0.031	>.05
WPF × E			−0.062	>.05	−0.073	>.05
Coping style × WPF × E					−0.103	.044
Adjusted R^2^	0.253		0.251		0.256	
ΔR^2]^	0.258	<.001	0.003	>.05	0.007	.044

β = standardized coefficient, E = extroversion, WPF = work-related psychological flexibility.

**Figure 2.**
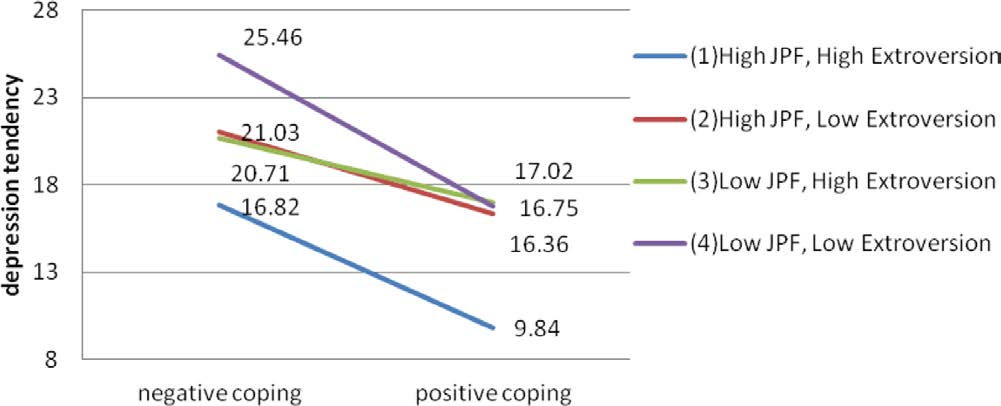
Interaction between coping style, job-related psychological flexibility, extroversion in depression tendency.

To better visualize the interaction of job-related psychological flexibility, coping style and extroversion personality in depression tendency, a 3-way interaction diagram was further constructed using the method as described previously elsewhere^[13]^ (Fig. 2). The degree of depression tendency was lower in healthcare workers with positive coping style than those with negative coping style for all combination of job-related psychological flexibility and extroversion. Healthcare workers with high job-related psychological flexibility, positive coping style and high extroversion had the lowest depression tendency scores. In contrast, those with low job-related psychological flexibility, negative coping style and low extroversion had the largest depression tendency scores. With taking positive coping style, individuals with high job-related psychological flexibility and high extroversion or low job-related psychological flexibility and low extroversion had more improved depression tendency compared to other 2 groups.

### 3.7. Interaction of coping style, job-related psychological flexibility and neuroticism in depression tendency

Similarly, 3 different models were constructed to investigate the interaction of coping style, job-related psychological flexibility and emotional stability in depression tendency in Chinese healthcare workers. The results are demonstrated in Table 4. In the model 1 including the main effects only, 43.0% variance of depression tendency was explained by the main effects. Coping style had a significantly negative association with depression tendency (*β* ;−0.193, *P < *.001), whereas neuroticism had a significantly positive association with depression tendency (*β = *0.503, *P < *.001). In the model 2 of the 2-way interaction terms between coping style, neuroticism and job-related psychological flexibility, the interaction terms did not show statistically significant. However, the main effects remained significant. In the model 3, the 3-way interaction term was added. The 3-way interaction term showed statistically significant association with depression tendency, and independently explained 1.0% of the variance in depression tendency (*β *= 0.116，*P < *.01). However, the main effects but not the 2-way interaction terms had significant association with depression tendency.

**Table 4:** Associations of WPF, neuroticism and coping style with depression tendency.

**Variable**	Model 1		Model 2		Model 3	
	β	*P* value	β	*P* value	β	*P* value
Coping style	−0.193	<.001	−0.193	<.001	−0.160	<.001
WPF	−0.138	<.001	−0.148	<.001	−0.118	.004
N	0.503	<.001	0.497	<.001	0.477	<.001
Coping style × WPF			−0.050	>.05	−0.038	>.05
Coping style × N			−0.053	>.05	−0.060	>.05
WPF × N			−0.040	>.05	−0.025	>.05
Coping style × WPF × N					0.116	.006
Adjusted R^2^	0.430		0.430		0.439	
ΔR^2^	0.433	<.001	0.005	>.05	0.010	.006

*β* = standardized coefficient, N = neuroticism, WPF = work-related psychological flexibility.

**Figure 3.**
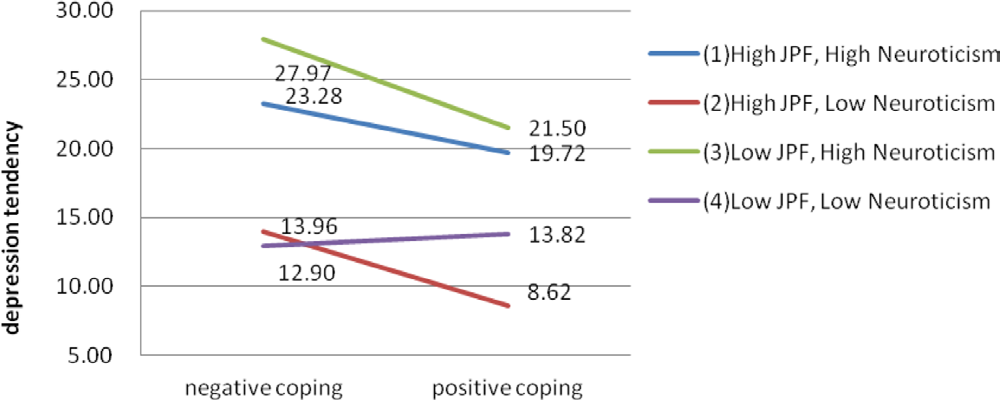
Interaction of Coping style, job-related psychological flexibility and neuroticism in depression tendency.

Figure 3 showed the interaction of coping style, job-related psychological flexibility and neuroticism in depression tendency. Chinese physicians with a positive coping style had a lower depression tendency than those with a negative coping style except those with low job-related psychological flexibility and low neuroticism. Individuals with low job-related psychological flexibility and high neuroticism had the highest depression tendency regardless of coping styles. However, with taking a positive coping style, the depression tendency was improved more in the individuals with low job-related psychological flexibility and high neuroticism or high job-related psychological flexibility and low neuroticism than others.

## 4. Discussion

In this study, we demonstrated that the associations of job-related psychological flexibility, coping style and personality types with and their interactions in depression tendency in Chinese healthcare workers. We found that extrovert unstable and negative coping style ranked the top risk factors for depression tendency, whereas extrovert stable and positive coping style ranked the top protective factors of depression tendency. This suggests that neuroticism (emotion unstable) is a risk factor of depression tendency. This is in the line with the previous findings. The tendency to experience emotion unstable constituted an important risk factor for depression.^[38]^ Individuals who had lack of cooperation and thinking introversion tended to exhibit depression.^[39]^ Further interaction analyses showed that a significant interaction of coping style, job-related psychological flexibility and personality types existed in depression tendency in Chinese healthcare workers. Particularly, with taking positive coping style, the depression tendency significantly declines at different rates in all individuals except those with low job-related psychological flexibility and low neuroticism. Moreover, we found that healthcare workers with high job-related psychological flexibility and high extroversion had the lowest depression tendency when they took the same coping style. These results extended the previous reports. There was an inverse relationship between low depression and the positive coping style.^[40]^ Psychological flexibility mediated the reductions in depression and stress.^[41]^ These observations suggest that depression tendency in healthcare workers are associated with personality, coping style and job-related psychological flexibility. Precision intervention strategies could be made to individually prevent depression in healthcare workers.

Personality types have been shown in association with depression.^[28]^ Individuals with stable emotion will not go to extremes by controlling their emotion even when they face tremendous stress in the environments. They always keep positive for life. In contrast, those with unstable emotion are more likely to have negative emotions, such as anger, anxiety and depression^[42]^ even when they experience a little changing situation. Generally, extrovert healthcare workers are optimistic and cheerful, easy to get along with colleagues, friends and relatives. They usually are brave enough to face challenges and solve problems with energetic, enthusiastic and open-minded regardless of the encouragement or help obtained from friends. Obviously, individuals with different personality types tend to adopt different coping styles. Coping style is a cognitive and behavioral strategy taken to manage internal and external demands of stressful events.^[43]^ In workplaces, positive coping style can lead to positive emotions and behaviors that improve mental outcomes.^[44]^ Thus, it is not surprising that coping styles are associated with depression in our study, and which is in the line with the previous study. When facing stress, a positive coping style is related to the lower levels of anxiety and depression, whereas a negative coping style is associated with higher levels of depression.^[45]^ Another previous study also showed that coping styles were associated with emotions and mental health in postdoctoral research fellows.^[46]^ Brain and colleagues reported that a positive coping style in solving problems enabled individuals to better adapt themselves to the society, while those taking a negative coping style in facing stress are susceptible to anxiety and depression.^[47]^

In job-related psychological flexibility, work acceptance is an important indicator. Individuals with a high work acceptance could have a psychological space for inner painful thoughts and feelings, and choose a meaningful life based on their life values.^[48]^ Even when they make some mistakes, or come across some difficulties, or face stress, they still can persist to pursue the goals and work effectively without being affected and worry. A high work acceptance always helps healthcare workers to make better use of the beneficial resources in the workplace from colleagues, friends, institutes, and society. A positive feedback is accompanied with a high work acceptance, which will make higher work commitment and rewards, realizing the value of life.^[48–50]^ Similarly, healthcare workers with a stronger work acceptance tend to take positive coping style when they face stress and difficulties, and are confident that they can complete the tasks without feeling too much pressure. Therefore, less chance of depression occurs, and they work more effectively with more mentally healthy. Thus, acceptance and commitment therapy is derived to prevent depression.

Limitations exist in this study. First, this survey was a cross-section study, the investigated factors may be accompanied rather than causal relationship. Thus, causal inference cannot be made from this study. Second, in this study, convenience sampling approach was applied to collect data, which is more convenient with a low cost, and a high response rate in comparison to random sampling. However, such sampling approach may have bias in representative, cautions should be paid in generalizing and interpreting the results. There are strengths, however, for this study. The sample size is relative large. High response and participant rates may overcome the disadvantage of convenience sampling. Longitudinal or intervention studies with a large sample size are warranted to investigate the causal relationships between psychological flexibility, coping style, personality types and depression.

In summary, we demonstrated that job-related psychological flexibility, positive coping style and extrovert stable personality were negatively associated with depression tendency in Chinese healthcare workers. Particularly, when taking positive coping style, depression tendency will be improved for most of the workers except those with low job-related psychological flexibility and low emotional stability. These findings suggest that a significant interaction of job-related psychological flexibility, coping style and personality types exists in depression tendency. Thus, enhancing the job acceptance and strengthening how to take active coping style may be able to reduce or prevent depression tendency in healthcare workers.

## Author contributions

YY and XJ designed the research, carried out data analysis, wrote the paper, and YY is the corresponding author. LL, YY and XJ provided help with the data analysis, interpretation, and write-up. All authors read and approved the final manuscript.

Conceptualization: Xiangzhi Jing.

Data curation: Xiangzhi Jing.

Formal analysis: Yongcheng Yao.

Funding acquisition: Yongcheng Yao.

Investigation: Yongcheng Yao.

Methodology: Lingeng Lu.

Project administration: Lingeng Lu.

Resources: Lingeng Lu.

Software: Lingeng Lu.

Supervision: Yongcheng Yao.

Validation: Lingeng Lu.

Visualization: Xiangzhi Jing.

Writing – original draft: Xiangzhi Jing.

Writing – review & editing: Lingeng Lu.

## Acknowledgments

We thank all participants who voluntarily completed the questionnaires in this study.
